# Association between Dairy Intake and Linear Growth in Chinese Pre-School Children

**DOI:** 10.3390/nu12092576

**Published:** 2020-08-25

**Authors:** Yifan Duan, Xuehong Pang, Zhenyu Yang, Jie Wang, Shan Jiang, Ye Bi, Shuxia Wang, Huanmei Zhang, Jianqiang Lai

**Affiliations:** Key Laboratory of Trace Element Nutrition of National Health and Family Planning Commission, National Institute for Nutrition and Health, Chinese Center for Disease Control and Prevention, No. 29 Nanwei Road, Xicheng District, Beijing 100050, China; duanyf@ninh.chinacdc.cn (Y.D.); pangxh@ninh.chinacdc.cn (X.P.); wangjie@ninh.chinacdc.cn (J.W.); jiangshan@ninh.chinacdc.cn (S.J.); biye@ninh.chinacdc.cn (Y.B.); wangsx@ninh.chinacdc.cn (S.W.); zhanghm@ninh.chinacdc.cn (H.Z.); jq_lai@126.com (J.L.)

**Keywords:** HAZ, stunting, dairy, pre-school children, cross-sectional study, China

## Abstract

Stunting remains a major public health issue for pre-school children globally. Dairy product consumption is suboptimal in China. The aim of this study was to investigate the relationship between dairy intake and linear growth in Chinese pre-school children. A national representative survey (Chinese Nutrition and Health Surveillance) of children aged under 6 years was done in 2013. Stratified multistage cluster sampling was used to select study participants. A food frequency questionnaire was used to collect dietary information. We calculated height-for-age Z-scores (HAZs) and estimated stunting using the 2006 WHO growth standard. In total, 12,153 children aged two to four years old (24 to <60 months) were studied from 55 counties in 30 provinces in China. Approximately 39.2% (4759/12,153) of those children consumed dairy at least once per day, 11.9% (1450/12,153) consumed dairy at least once in the last week, and nearly half (48.9%, 5944/12,153) did not have any dairy in the last week. The HAZ was −0.15 ± 1.22 and the prevalence of stunting was 6.5% (785/12,153). The HAZ for children who consumed dairy at least once per day or per week was 0.11 points or 0.13 points higher than the children without dairy intake. The risk of stunting for children who consumed dairy at least once per day was 28% lower than the children without dairy intake in the last week, and the risk was similar between weekly dairy consumption and no dairy consumption (AOR: 1.03, 95% CI: 0.74–1.42) after adjusting for potential confounders, including socioeconomic characteristics, lifestyle, health status, and the intake frequency of other foods. Dairy intake was significantly associated with a higher HAZ and a lower risk of stunting for children aged 2–4 years old in China. The proportion of dairy intake was still low in Chinese pre-school children. The promotion of dairy consumption might be an effective and feasible measurement for improving linear growth in Chinese pre-school children.

## 1. Introduction

Linear growth is the best overall indicator of children’s well-being and should be promoted for assessing nutritional status, designing programs, and assessing impacts [1,2,3]. Stunting is the most prevalent form of child malnutrition worldwide. Stunting is associated with increased morbidity and mortality, loss of physical growth potential, neurodevelopmental and cognitive function retardation, and an elevated risk of chronic diseases in adulthood [4]. Despite the slow reduction in recent years, stunting still affected an estimated 21.3% or 144 million children under five years old globally in 2019 [5]. One of the six global nutrition targets is to reduce the number of stunted children under 5 years of age by 40% before 2025 [6]. At the current rate of progress, it will be a challenge to achieve the global goal of decreasing the number of stunted children to 100 million in 2025 [4]. Asia is the continent with the most stunted children globally, which has more than half of all stunted children under five years old, with an estimated 78.2 million in 2019 [5].

As an optimal source of nutrients and bioactive factors, milk and dairy products play an important role in childhood growth and development [7]. Several studies have investigated the association between dairy intake and linear growth since the 1920s [8,9]. However, the conclusions are inconsistent in both the observational and interventional studies. In one of the first studies in 1928, Orr et al. estimated an increase in height of 20% for Scottish children aged 5–14 years who consumed milk in addition to their normal diet for seven months in comparison to children who did not [8]. An early study in 1978 found significant increases in height for children consuming milk compared to a control group in a school milk intervention in New Guinea [10]. A recent randomized controlled trial in Vietnam showed that the height-for-age Z-score (HAZ) significantly improved over 6 months of milk intervention in 454 children aged 7–8 years and stunting dropped by 10% [11], while another randomized controlled trial found no significant change in HAZs in Kenyan school children [12].

Dairy consumption is generally low in the Chinese population. Although dairy consumption increased from 1982 to 2002 in the Chinese population and reached a peak in 2002, there was a pronounced decline in the following decade [13]. In 2010, the amount of dairy consumption by Chinese people was 24.9 g per day, most of which was liquid cow milk. Children aged 2–3 years old took part in higher dairy consumption (≈80 g per day) than 4–6-year-old children (≈45 g per day) did. Even for 2–3-year-old children, only 4.3% of them achieved the daily dairy consumption recommendation and this proportion decreased to 1.1% in 4–6-year-old children [13]. Cow’s milk allergy and lactose intolerance may contribute to low dairy consumption to some extent in the Chinese population. Cow’s milk allergy is one of the most common food allergies, with an estimated prevalence in developed countries ranging from 0.5–3% at 1 year of age [14]. Gupta et al. estimated the overall prevalence of cow’s milk allergy was 1.7% (95% confidence interval: 1.5–1.8%) and peaked in children aged 0–5 years at 2.0% [15]. Approximately 20% of Hispanic, Asian, and Black children younger than 5 years of age display evidence of lactase deficiency and lactose malabsorption [16]. In China, a study showed that the incidences of lactase deficiency and lactose intolerance were 38.5% and 12.2% in 3–5-year-old children, respectively [17].

The prevalence of stunting was high in Chinese children under 6 years old, especially in poor rural areas [18]. Among other reasons for stunting, inadequate animal-sourced food intake is a critical risk factor for the stunting of children under 5 years old [19], of which dairy consumption was associated with higher HAZs of children aged 6–23 months [20]. The first 1000 days is considered the crucial period for correcting child stunting. Few studies have focused on the effects of dairy consumption on the linear growth of pre-school children in developing countries. In China, low dairy intake and the high prevalence of stunting in pre-school children co-existed. This study aimed to investigate the relationship between dairy intake and linear growth in Chinese pre-school children.

## 2. Materials and Methods

### 2.1. Study Design

The study was a secondary data analysis from a nationally representative survey (Chinese Nutrition and Health Surveillance (CNHS)) in 2013 and a subpopulation was selected from the original study. The detailed methods of the CNHS were described previously [18]. Briefly, this was a cross-sectional survey among children under 6 years of age and lactating mothers from 30 provinces, autonomous regions, and municipalities in mainland China (the Tibet Autonomous Region was not included in the survey). Multi-stage stratified cluster random sampling was used in the study. In total, 2865 districts/counties in China were categorized into four strata (large cities, medium and small cities, non-poor rural areas, and poor rural areas) based on the population size and the definition of urban or rural from the National Bureau of Statistics of the People’s Republic of China. A city with a population size of more than 1,000,000 was defined as a large city, and other cities belonged to medium and small cities category. Poor rural areas were the key counties for poverty alleviation and development identified by the Framework for Poverty Alleviation and Development in Chinese Rural Areas, while other counties belonged to the non-poor rural areas. Then, 55 counties (12 metropolises, 15 medium and small cities, 18 non-poor rural areas, and 10 poor rural areas) were chosen in the study. In each selected county, three communities/townships were systematically sampled. In each selected township, three neighborhoods/villages were systematically selected. Finally, 10 children from each age group were randomly selected in each selected village. The total sample size was 34,650 for children under 6 years old, of which, 14,850 were children aged two to four years old (24 to <60 months). The sample size calculation was based on an estimation of the prevalence of anemia in children under 6 years old after taking the complex sampling design into account.

### 2.2. Subjects

Children were selected according to three age groups (24–35 months, 36–47 months, and 48–59 months). Their caregivers were asked to finish a face-to-face interview with well-trained staff. The Ethics Review Board of the National Institute for Nutrition and Health, Chinese Center for Disease Control and Prevention, approved the protocol (No. 2013–018). All caregivers gave their informed consent in writing to participate before starting the interview.

### 2.3. Data Collection

The socioeconomic, family care, dietary intake, lifestyle, and health-related information were collected using the questionnaires during the face-to-face interviews mentioned above. The caregivers of children older than 2 years old were asked to finish a food frequency questionnaire (FFQ) in order to collect the dietary information regarding the past week before the survey, which was modified from a Chinese food frequency questionnaire established by Zhao et al. [21], who also examined the validity and reliability. The FFQ questionnaire, which was adopted in this survey, consisted of 44 food and beverage items, and was categorized into 10 food groups (1. cereal grains, roots, and tubers; 2. legumes and legume products; 3. dairy products from cows, goats, buffalo, etc. (whole milk, skim milk, milk powder, infant formula, yogurt, cheese); 4. flesh foods (meat, fish, poultry, and liver/organ meats); 5. eggs; 6. vegetables; 7. fruits; 8. snacks; 9. beverages; 10. nuts). The frequency of dairy product consumption was divided into three levels for this study. Eating at least once per day in the week before the survey was defined as “daily” consumption. “Weekly” consumption means the food was eaten once or more in the last week but less than once per day. If there was no consumption of the food or the frequency was less than once in the last week, the frequency was named “none.”

### 2.4. Primary Outcomes

The standing height of children was measured by well-trained staff using a stadiometer with an accuracy of 0.1 cm. The height measurement was asked to be without the children having braided hair or shoes on. The primary outcome measures were the height-for-age Z-scores (HAZs) and the prevalence of stunting. Z-scores were calculated using the WHO Anthro software (WHO Anthro for personal computers, version 3.2.2, World Health Organization, Geneva, Switzerland.) Values were expressed as SD scores (Z-scores) using the reference population of the 2006 WHO growth standard [22]. The conversion of anthropometric variables to sex- and age-specific Z-scores were performed using the WHO standard. Stunting was defined as a height-for-age Z-score less than −2 SD of the median height of the WHO reference population.

### 2.5. Statistical Analysis

Data were entered via a standardized data management platform and were cleaned for all variables. All the data were analyzed using SAS 9.4 software (SAS Institute Inc., Cary, NC, USA). The frequency of consumption of each food group, including dairy, was tested using correlation analysis, which showed that each of the other food groups was uncorrelated with dairy consumption. Therefore, the frequency of consumption of each food group was viewed as an independent variable in the model. HAZs were expressed as mean ± SD and the differences were compared using ANOVA tests. The multivariate linear regression analysis was used to assess the relationship between HAZs and the frequency of dairy consumption after controlling for the potential confounders (e.g., residential area, children’s age group, ethnicity, parental education level, parental age and occupation, birth weight and length, major caretaker, duration of daytime outdoors, regular growth monitoring, and the frequency of egg consumption). Categorical variables were expressed as a percentage (%). Chi-square tests were used for categorical variable comparisons. The logistic procedure was used to assess the relationship between stunting prevalence and the frequency of dairy consumption after controlling for the potential confounders (e.g., residential area, ethnicity, maternal occupation and migrant status, birth weight, major caretaker, sleep duration, regular growth monitoring, incidence of respiratory system disease in the last two weeks, and frequency of egg and fruit consumption). First, a bivariate analysis was conducted between each linear growth indicator and each potential confounder. The variables marginally associated with outcome variables in the bivariate analysis were selected for multivariate analyses (*p* < 0.20). Then, a multivariate analysis was conducted for each linear growth indicator with potential confounders. In the final model, only variables significantly associated with outcome variables were retained (*p* < 0.05). The adjusted β value and standard error (SE) were reported in the final linear regression model while retaining all significant variables, and the adjusted odds ratio (OR) and 95% confidence intervals (CIs) were reported in the final logistic regression model while retaining all significant variables.

## 3. Results

In total, 12,153 children aged two to four years old (24 to <60 months) were included in the study, where 51.5% (6261/12,153) of the children were boys and 48.9% of the children lived in an urban area. Approximately 39.2% (4759/12,153) of the children consumed dairy at least once per day, 11.9% (1450/12,153) of them consumed dairy at least once in the past week, and nearly a half (48.9%, 5944/12,153) of them did not have any dairy in the past week. The average height-for-age Z-score (HAZ) was −0.15 ± 1.22 and the prevalence of stunting was 6.5% (785/12,153).

The residential area, ethnicity, parental education status, parental age, occupation, migrant status, and household income was significantly associated with the HAZ and stunting in the bivariate analysis (*p* < 0.001) (Table 1). The birth weight and length, incidence of respiratory system disease in the last two weeks, major caretaker, regular growth monitoring, duration of daytime outdoors, and frequency of egg or fruit consumption was significantly associated with the HAZ and stunting in the bivariate analysis (*p* < 0.001) (Table 2).

According to the results of the bivariate analysis, which are listed in Table 1 and Table 2, the variables marginally associated with the HAZ or the prevalence of stunting in the bivariate analysis were selected for multivariate analyses (*p* < 0.20). In the final model, only variables significantly associated with outcome variables were retained (*p* < 0.05).

After adjusting for residential area, children’s age group, ethnicity, parental education level, parental age and occupation, birth weight and length, major caretaker, duration of daytime outdoors, regular growth monitoring, and the frequency of egg consumption, the HAZ was significantly associated with the frequency of dairy intake. The HAZ was 0.11 points or 0.13 points greater for children who consumed dairy at least once per day or per week, respectively, than the children without dairy intake in the past week (Table 3).

After adjusting for residential area, ethnicity, maternal occupation and migrant status, birth weight, major caretaker, sleeping duration, regular growth monitoring, incidence of respiratory system disease in the last two weeks, and frequency of egg and fruit consumption, the children with daily dairy consumption had a 28% lower risk of stunting than the children without dairy intake in the past week. Meanwhile, the risk of stunting was similar between weekly dairy consumption and without dairy consumption in the past week (Table 4).

## 4. Discussion

Dairy consumption was quite low in Chinese pre-school children, where nearly half of the children did not consume dairy during the past week. Dairy intake was significantly associated with greater height-for-age Z-scores and a lower risk of stunting for children aged 2–4 years old in China.

Linear growth failure was common for 2–4-year-old Chinese children, especially in poor rural areas. Although the etiology of stunting is poorly understood, prenatal and postnatal nutritional deficits could contribute to the stunting of children under 5 years of age [23]. Inadequate intake of one or more nutrients, including energy, protein, and micronutrients, such as zinc, vitamin A, and phosphorus, may result in the growth retardation of children. In addition, repeated infections worsen nutrient deficiency and impair the absorption of nutrients [24].

Dairy consumption in childhood has long been assumed to be beneficial for growth, which was proposed as the “milk hypothesis” by Bogin [25]. The specific stimulating effect of milk on linear growth may be related to several components of milk, such as high-quality protein, bioactive peptides, amino acids, insulin-like growth factor-1 (IGF-1), or minerals, including calcium. Dairy products provide high-quality protein with peptides and bioactive factors that could have specific effects on growth. A prospective cohort found that dairy protein intake was a significant predictor of peak height velocity and adult height, while animal or vegetable protein was not [26]. Approximately 80% of the protein in cow’s milk is casein, and the remaining 20% is whey [7]. Whey, as a soluble milk protein, may have some insulinotropic components [27]. Another study conducted on eight-year-old boys suggested that casein might have a stronger IGF-1 stimulating effect than whey does [28].

Apart from proteins, milk IGF-1 is another major relevant factor for children’s development and growth. Milk IGF-1 is structurally identical to human IGF-1 and the milk IGF-1 concentration is approximately 30 ng/mL [29]. IGF-1 is a potential growth factor in bone and mediates the effects of the pituitary growth hormone [30]. IGF-1 facilitates bone growth by increasing the uptake of amino acids, which are then integrated into new proteins in bone tissue [31]. It is the most abundant growth factor in bone and has a strong anabolic effect on growing bone tissue since it stimulates the chondrocytes in the epiphyseal plate [32]. Serum levels of IGF-1 rise after milk consumption, although it is not clear whether this is due to the IGF-1 in milk or whether milk consumption stimulates endogenous IGF-1 production [33]. In studies of children, milk consumption, circulating IGF-1, and height were positively correlated [7,34,35], and milk supplementation resulted in an elevation of IGF-1 levels [7,36,37]. Thus, milk may promote linear growth through an IGF-1-mediated process, perhaps in concert with calcium or other milk constituents [30].

With respect to linear growth, the effect of calcium was unclear. Some calcium supplementation or calcium fortification studies did not appear to positively influence height in children [38,39,40,41,42]. However, a study with a sample of 1002 children aged 24–59 months from the National Health and Nutrition Examination Survey (NHANES) showed that total calcium intake was positively associated with height and it might mediate the relationship between milk intake and height. The author hypothesized that calcium appears to play a role in the increased height of young children, but it may act synergistically with other components of milk [30].

The lactase persistence (LP) phenotype was studied as a proxy for dairy intake in some studies in recent years. So far, the relationship between the LP phenotype and milk consumption is inconclusive. Some studies have found that milk or total dairy consumption is associated with the LP genotype [43,44,45,46,47]. Other studies have shown that LP genetics has no influence over whether a participant is a cow milk consumer [48,49,50]. Most of those studies were conducted in populations with high frequencies of the LP phenotype and high amounts of milk consumption. A study showed that the incidence of lactase deficiency was 38.5% and the proportion of lactose intolerance was 12.2% in Chinese 3–5-year-old children [17].

The relationship between dairy intake and height is inconclusive for children. A systematic review and meta-analysis of controlled trials assessed the effects of supplementing a usual diet with dairy products on physical growth, including twelve studies conducted in Europe, USA, China, Northern Vietnam, Kenya, Indonesia, and India between the 1920s and 2000s. Only one of these studies was conducted in preschool children in Beijing suburbs; the others were all conducted in school-aged children from 7–13 years old. The meta-analysis with a random effects model yielded a pooled estimate of 0.59 cm. This additional growth was the result of giving a daily milk supplement of 245 mL for 12 months on average. In addition, the results of the sensitivity analysis suggested an effect size of 0.4 cm could be considered a conservative estimate [51]. The protective effect against stunting was also found in a study involving 68 low- and middle-income countries, which suggested that milk consumption is associated with a reduced probability of being stunted of 1.9 percentage points for children aged 6–59 months. This study showed that a child aged 24 to 59 months that consumed milk had a 0.14 points greater HAZ (*p* < 0.001) than those that did not consume milk [52], which was in accordance with our study. Despite these results, the relationship may be different in the populations who consumed different quantities of dairy. Wiley found that children in the highest quartile of milk intake were taller (1.1–1.2 cm, *p* < 0.01) than those in the middle quartiles. Interestingly, this difference was not found between the group in the highest quartile of milk drinkers and the lowest quartile. Furthermore, this study also evaluated the association between milk consumption and height among preschool-age children in the USA, 89% of whom reported daily dairy consumption. Results showed that children who drank milk daily were 1.0 cm taller (*p* < 0.02) than those with a less frequent intake [30].

As mentioned above, intervention studies that focus on preschool children are scarce. Although growth was relatively stable during the preschool years, the growth velocity is still high in this period. This period should be viewed as a sensitive life stage for intervention. Interventions beyond 24 months that prove successful in enhancing adult stature (especially in girls) may offer additional opportunities to improve nutritional status and would likely foster advantages throughout the mothers’ entire reproductive life and benefit future generations [53].

This study has some limitations. First, the CNHS is a cross-sectional survey; as such, causality cannot be attributed to dairy in this study design. Second, the amount of dairy intake was not included in our study. However, the FFQ is valid and with good reliability for assessing preschool children’s food intake [54]. Furthermore, the frequency of dairy intake can be recalled by subjects more easily and accurately than the amount of dairy intake and the results can be modifiable for education and intervention design. Third, since the frequency was recalled over the past week, it was only a snapshot of the dairy consumption of children. This snapshot may or may not be reflective of the overall patterns of dairy consumption.

## 5. Conclusions

The amount of dairy intake remains low in Chinese pre-school children. The dairy intake was positively associated with linear growth in pre-school children in China. A more frequent dairy intake might promote a greater HAZ and a lower prevalence of stunting in Chinese pre-school children. The promotion of dairy consumption might be an effective and feasible measurement for improving linear growth in pre-school children. Further intervention studies are warranted to test the relationship.

## Figures and Tables

**Table 1:** The relationship between socioeconomic status and height-for-age Z-score (HAZ) and the prevalence of stunting.

Variables	% (*n*/*N*)	HAZ (Mean ± SD)	*p*-Value ^1^	Prevalence of Stunting (%)	*p*-Value ^2^
Residential area					
Urban—metropolis	21.1% (2561/12,153)	0.28 ± 1.12	<0.001	2.0	<0.001
Urban—middle or small cities	27.9% (3386/12,153)	0.01 ± 1.15		4.0	
Rural—non-poor areas	32.9% (3997/12,153)	−0.20 ± 1.16		6.0	
Rural—poor areas	18.2% (2209/12,153)	−0.82 ± 1.23		16.3	
Age group (years)					
2~	31.8% (3858/12,153)	−0.11 ± 1.27	0.016	7.0	0.076
3~	33.5% (4073/12,153)	−0.18 ± 1.22		6.7	
4~	34.7% (4222/12,153)	−0.17 ± 1.17		5.8	
Gender					
Male	51.5% (6261/12,153)	−0.14 ± 1.24	0.083	6.8	0.148
Female	48.5% (5892/12,153)	−0.17 ± 1.19		6.1	
Ethnicity					
Han	85.1% (10,346/12,153)	−0.06 ± 1.19	<0.001	4.9	<0.001
Minority	14.9% (1807/12,153)	−0.71 ± 1.26		15.2	
Maternal education					
Primary or below	15.3% (1783/11,695)	−0.63 ± 1.19	<0.001	11.8	<0.001
Junior high	49.2% (5750/11,695)	−0.27 ± 1.20		7.4	
Senior high or above	35.6% (4162/11,695)	0.23 ± 1.15		2.7	
Paternal education					
Primary or below	11.1% (1274/11,501)	−0.71 ± 1.21	<0.001	13.8	<0.001
Junior high	49.3% (5672/11,501)	−0.30 ± 1.19		7.5	
Senior high or above	39.6% (4555/11,501)	0.19 ± 1.15		3.1	
Maternal age group (years)					
≤26	20.7% (2421/11,708)	−0.35 ± 1.23	<0.001	9.0	<0.001
27–30	31.2% (3650/11,708)	−0.14 ± 1.22		6.3	
31–34	25.6% (2991/11,708)	−0.04 ± 1.19		5.2	
≥35	22.6% (2646/11,708)	−0.10 ± 1.21		5.6	
Paternal age group (years)					
≤28	23.0% (2643/11,516)	−0.23 ±1.23	<0.001	8.0	0.003
29–32	28.1% (3233/11,516)	−0.12 ± 1.22		5.9	
33–36	23.5% (2706/11,516)	−0.11 ± 1.20		5.9	
≥37	25.5% (2934/11,516)	−0.15 ± 1.21		6.1	
Maternal occupation					
Unemployed	34.8% (4072/11,689)	−0.20 ± 1.19	<0.001	6.3	<0.001
Farmer	17.1% (1999/11,689)	−0.63 ± 1.24		13.6	
Others	48.1% (5618/11,689)	0.06 ± 1.18		4.0	
Paternal occupation					
Unemployed	6.1% (701/11,526)	−0.43 ± 1.19	<0.001	9.4	<0.001
Farmer	23.1% (2660/11,526)	−0.56 ±1.25		12.5	
Others	70.8% (8165/11,526)	0.00 ± 1.17		4.3	
Annual household income (per capita CNY *)					
≥15,000	35.3% (4282/12,147)	0.03 ± 1.18	<0.001	4.4	<0.001
10,000–14,999	17.7% (2155/12,147)	−0.16 ± 1.19		6.0	
5000–9999	19.9% (2420/12,147)	−0.31 ± 1.25		8.8	
<5000	16.8% (2035/12,147)	−0.47 ± 1.25		10.3	
refuse	10.3% (1255/12,147)	0.03 ± 1.15		3.6	
Maternal migrant status					
Migrant mother	15.0% (1824/12,153)	−0.45 ± 1.22	<0.001	10.8	<0.001
Mother living at home	85.0% (10,329/12,153)	−0.10 ± 1.21		5.7	
Paternal migrant status					
Migrant father	24.0% (2919/12,153)	−0.36 ± 1.22	<0.001	8.7	<0.001
Father living at home	76.0% (9234/12,153)	−0.09 ± 1.21		5.7	

^1^ The result of the variance analysis between the variable and the HAZ. ^2^ The result of the chi-square test between the variable and the prevalence of stunting. * CNY: Chinese Yuan.

**Table 2:** The relationship between the health status and lifestyles and the HAZ and the prevalence of stunting.

Variables	% (*n*/*N*)	HAZ (Mean ± SD)	*p*-Value ^1^	Prevalence of Stunting (%)	*p*-Value ^2^
Birth weight (g)					
<2500	3.6% (436/12,142)	−0.64 ± 1.27	<0.001	12.6	<0.001
2500–3200	41.3% (5013/12,142)	−0.33 ± 1.19		8.0	
3201–3999	40.4% (4909/12,142)	0.06 ± 1.18		4.2	
≥4000	7.5% (915/12,142)	0.29 ± 1.18		3.4	
Unknown	7.2% (869/12,142)	−0.54 ± 1.25		10.6	
Birth length (cm)					
<50	12.8% (1549/12,133)	−0.23 ± 1.17	<0.001	6.7	<0.001
=50	36.4% (4410/12,133)	−0.01 ± 1.15		4.5	
>50	19.5% (2365/12,133)	0.18 ± 1.23		4.1	
Unknown	31.4% (3809/12,133)	−0.50 ± 1.22		10.1	
Premature					
Yes	9.9% (1179/11,958)	−0.21 ± 1.16	0.055	6.0	0.656
No	90.1% (10,779/11,958)	−0.14 ± 1.22		6.4	
Incidence of respiratory system disease in the last two weeks					
Yes	24.5% (2973/12,109)	−0.06 ± 1.15	<0.001	4.3	<0.001
No	75.5% (9136/12,109)	−0.18 ± 1.24		7.2	
Incidence of diarrhea in the last two weeks					
Yes	4.9% (597/12,122)	−0.15 ± 1.10	0.997	4.7	0.073
No	95.1% (11,525/12,122)	−0.15 ± 1.22		6.5	
Major caretaker					
Grandmothers	18.0% (2187/12,153)	−0.30 ± 1.20	<0.001	8.4	<0.001
Mother and father	42.9% (5219/12,153)	−0.07 ± 1.23		5.7	
Mother	36.8% (4470/12,153)	−0.16 ± 1.21		6.1	
Father	1.6% (198/12,153)	−0.58 ± 1.23		10.6	
Others	0.7% (79/12,153)	−0.57 ± 1.24		15.2	
Regular growth monitoring					
Yes	72.6% (8819/12,141)	−0.01 ± 1.18	<0.001	4.8	<0.001
No	27.4% (3322/12,141)	−0.52 ± 1.23		10.8	
Sleep duration (hours)					
<10	13.8% (1678/12,141)	−0.23 ± 1.26	0.010	7.6	<0.001
10 to <10.5	33.8% (4104/12,141)	−0.15 ± 1.19		5.4	
10.5 to <12	25.5% (3098/12,141)	−0.11 ± 1.19		6.2	
≥12	26.9% (3261/12,141)	−0.16 ± 1.25		7.5	
Duration of daytime outdoors (minutes)					
≤90	25.1% (3049/12,134)	0.03 ± 1.24	<0.001	5.1	<0.001
91–150	26.8% (3257/12,134)	−0.06 ± 1.20		5.7	
151–240	34.7% (4212/12,134)	−0.27 ± 1.21		7.3	
>240	13.3% (1616/12,134)	−0.37 ± 1.17		8.6	
Have been breastfed in the last 24 h					
Yes	1.4% (164/12,136)	−0.24 ± 1.30	0.355	9.8	0.083
No	98.7% (11,972/12,136)	−0.15 ± 1.22		6.4	
Cow‘s milk allergy					
Yes	0.8% (93/12,081)	−0.27 ± 1.27	0.344	8.6	0.388
No	99.2% (11,988/12,081)	−0.15 ± 1.22		6.4	
Frequency of egg consumption					
Daily	36.0% (4369/12,153)	0.11 ± 1.17	<0.001	3.8	<0.001
Weekly	48.5% (5898/12,153)	−0.23 ±1.20		6.7	
None	15.5% (1886/12,153)	−0.52 ± 1.23		11.8	
Frequency of fruit consumption					
Daily	56.1% (6812/12,153)	−0.01 ± 1.18	<0.001	4.6	<0.001
Weekly	38.3% (4657/12,153)	−0.33 ± 1.23		8.4	
None	5.6% (684/12,153)	−0.43 ± 1.34		11.8	

^1^ The result of the variance analysis between the variable and the HAZ. ^2^ The result of the chi-square test between the variable and the prevalence of stunting.

**Table 3:** Association between the HAZ and the frequency of dairy consumption.

Frequency of Dairy Consumption	HAZ (Mean ± SD)	β ^#^	SE	*t*	*p*-Value
Daily	0.13 ± 1.14	0.11	0.03	4.23	<0.001
Weekly	−0.01 ± 1.16	0.13	0.04	3.64	<0.001
None	−0.42 ± 1.23	Ref.	-	-	-

Ref. means the reference group. ^#^ Adjusted by residential area, children’s age group, ethnicity, parental education level, parental age and occupation, birth weight and length, major caretaker, duration of daytime outdoors, regular growth monitoring, and the frequency of egg consumption.

**Table 4:** Association between the prevalence of stunting and the frequency of dairy consumption.

Frequency of Dairy Consumption	Prevalence of Stunting (%)	Crude OR (95% CI)	Adjusted OR ^#^ (95% CI)	*p*-Value
Daily	3.2	0.32 (0.26, 0.38)	0.72 (0.58,0.90)	0.003
Weekly	4.1	0.78 (0.57, 1.05)	1.03 (0.74, 1.42)	0.875
None	9.6	1.00	1.00	-

^#^ Adjusted by residential area, ethnicity, maternal occupation and migrant status, birth weight, major caretaker, sleeping duration, regular growth monitoring, incidence of respiratory system disease in the last two weeks, and the frequency of egg and fruit consumption.
